# Quantitative analysis of intestinal perfusion with indocyanine green (ICG) and methylene blue (MB) using a single clinically approved fluorescence imaging system: a demonstration in a porcine model

**DOI:** 10.1007/s00464-024-10864-1

**Published:** 2024-05-10

**Authors:** Danique J. I. Heuvelings, Max H. M. C. Scheepers, Zaid Al-Difaie, Nariaki Okamoto, Michele Diana, Laurents P. S. Stassen, Nicole D. Bouvy, Mahdi Al-Taher

**Affiliations:** 1 NUTRIM School of Nutrition and Translational Research in Metabolism, Maastricht University, Maastricht, The Netherlands; 2 Department of Surgery, Maastricht University Medical Center, Maastricht, The Netherlands; 3 GROW School for Oncology and Developmental Biology, Maastricht University, Maastricht, The Netherlands; 4 IRCAD, Research Institute Against Digestive Cancer, Strasbourg, France; 5 ICube Laboratory, Photonics Instrumentation for Health, Strasbourg, France; 6 Department of Digestive and Endocrine Surgery, University Hospital of Strasbourg, Strasbourg, France

**Keywords:** Bowel perfusion assessment, Methylene blue, Indocyanine green, Intraoperative near-infrared fluorescence imaging, Anastomotic leakage, Bile duct imaging

## Abstract

**Background:**

Near-infrared fluorescence (NIRF) angiography with intraoperative administration of indocyanine green (ICG) has rapidly disseminated in clinical practice. Another clinically approved, and widely available dye, methylene blue (MB), has up to now not been used for this purpose. Recently, we demonstrated promising results for the real-time evaluation of intestinal perfusion using this dye. The primary aim of this study was to perform a quantitative analysis of bowel perfusion assessment for both ICG and MB.

**Methods:**

Four mature female Landrace pigs underwent laparotomy under general anesthesia. An ischemic bowel loop with five regions of interest (ROIs) with varying levels of perfusion was created in each animal. An intravenous (IV) injection of 0.25 mg/kg–0.50 mg/kg MB was administered after 10 min, followed by NIRF imaging in MB mode and measurement of local lactate levels in all corresponding ROIs. This procedure was repeated in ICG mode (IV dose of 0.2 mg/kg) after 60 min. The quest spectrum fluorescence camera (Quest Medical Imaging, Middenmeer, The Netherlands) was used for NIRF imaging of both MB and ICG.

**Results:**

Intraoperative NIRF imaging of bowel perfusion assessment with MB and ICG was successful in all studied animals. Ingress (i/s) levels were calculated and correlated with local lactate levels. Both MB and ICG ingress values showed a significant negative correlation (*r* = − 0.7709; *p* =  < 0.001; *r* = − 0.5367, *p* = 0.015, respectively) with local lactate levels. This correlation was stronger for MB compared to ICG, although ICG analysis showed higher absolute ingress values.

**Conclusion:**

Our fluorescence quantification analysis validates the potential to use MB for bowel perfusion assessment besides the well-known and widely used ICG. Further human studies are necessary to translate our findings to clinical applications.

**Supplementary Information:**

The online version contains supplementary material available at 10.1007/s00464-024-10864-1.

Anastomotic leakage (AL) is a highly concerning complication, which can occur after colorectal surgery with anastomosis formation. Impaired blood flow to the bowel is considered the primary factor contributing to AL. Consequently, bowel perfusion assessment is a critical approach to reduce the occurrence of AL [1, 2]. Intraoperative near-infrared fluorescence imaging (NIRF), using administration of an optical dye, offers a convenient and versatile method to improve the assessment of anastomotic perfusion. Indocyanine green (ICG) is the dye that is most commonly used for this purpose as it provides favorable results [3–7]. Recently, our research team demonstrated in an experimental study that methylene blue (MB), another widely available dye, showed promising results too [8]. As it is partly cleared by the kidneys, it was previously demonstrated that it could successfully visualize the ureters intraoperatively. Less expected was the demonstration that it could also be used for intraoperative perfusion imaging, reflecting a potential benefit in comparison to ICG that is exclusively cleared by the liver and subsequently cannot visualize the ureters non-invasively.

Our promising results with MB were gathered using a commercially available NIRF imaging system, which can visualize both dyes due to its bimodal properties (QUEST SPECTRUM®, Quest Medical Imaging, Middenmeer, The Netherlands). Since this NIRF imaging system uses two different wavelength modes, it solves one of the drawbacks related to the use of MB, namely its excitation characteristics. MB has an excitation peak of about 700 nm, an excitation wavelength of 668 nm, and an emission of 688 nm which can be seen with the naked eye [9]. These characteristics are different from ICG that is excited at around 800 nm, until recently requiring a different imaging system. So far, the majority of imaging systems used in MB studies were experimental and not commercially accessible for clinical purposes conversely to the system used in the present study. As the Federal Drug Administration (FDA) and the European Medicines Agency (EMA), have approved the clinical use of MB as well [9], this dye may be promising to use for multipurpose NIRF imaging. Our research team therefore considered it clinically relevant to investigate the use of MB for bowel perfusion assessment.

The objective of the current study was to conduct a quantitative analysis of bowel perfusion assessment using a commercially available NIRF imaging system, comparing the visualization obtained with MB and ICG.

## Materials and methods

This study was performed at the central animal facilities of Maastricht University (Maastricht, The Netherlands) and was approved by the local Experimental Animal Committee (DEC) (2017-021-001). All animals were used in compliance with Dutch regulations and legislation concerning animal research.

### Animals and anesthesia

Four mature female Landrace pigs (35–45 kg) underwent general anesthesia ensured with appropriate analgesia using the following medication: intramuscular injection of Zolazepam/Tiletamine (6 mg/kg, Virbac, Barneveld, The Netherlands) and Thiopental (10 mg/kg, Panpharma SA, Trittau, Germany), a combination of sufentanyl (0.01 mg/kg/h, Hameln Pharma GmbH, Hameln, Germany), Propofol (9 mg/kg/h, B. Braun Melsungen AG, Melsungen, Germany), and Midazolam (1 mg/kg/h, Aurobindo, Baarn, The Netherlands) intravenously. All pigs were intubated and mechanically ventilated. Alterations in vital parameters were monitored by an animal anesthesiologist, and whenever necessary, anesthesia and analgesia were intensified. At the end of the procedure, all animals were euthanized with a lethal dose of 200 mg/kg pentobarbital.

### Preparation of dyes

The preparation of dyes was carried out as previously described [8]. In short, MB was diluted in a sterile phosphate-buffered saline (PBS) solution to a concentration of 1 mg/mL and ICG in a sterile H_2_O solution to a concentration of 2.5 mg/mL. An IV dose of 0.25 or 0.50 mg/kg of MB (doses based on a previously published dose finding study [8]) and 0.2 mg/kg of ICG was administered. This ICG dose is the current frequently clinically used dose range in patients, based on the analysis of 1,240 patients registered in the EURO-FIGS registry on fluorescence angiography [10].

### Surgical procedure and measurements

**Fig. 1:**
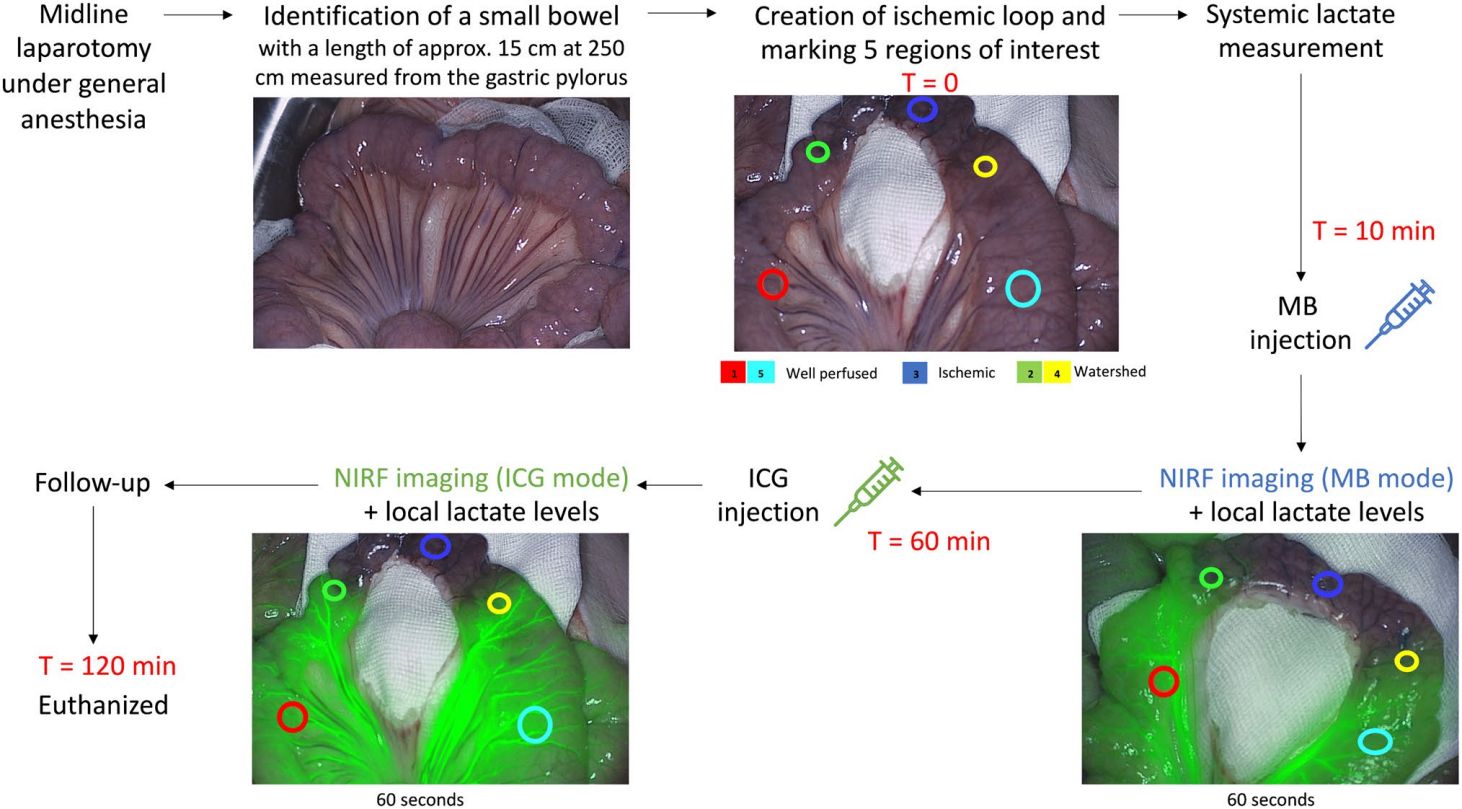
Schematic overview of the surgical procedure and measurements. *T* = 0 represents the timepoint at which the ischemic loop was created. After 10 min (*T* = 10) MB was injected. One hour after the creation of the ischemic bowel loop (*T* = 60), ICG was injected. NIRF imaging continued until at least 120 min

After appropriate sedation and analgesia, a midline laparotomy was performed by an experienced surgeon. A small bowel loop with a length of approximately 15 cm, was measured at 250 cm from the gastric pylorus. To ensure optimal exposure, the loop was placed on a gauze. The mesenteric side with at least 8 vessels was transected to create a gradual ischemic loop, as described in an earlier study [11]. After compromising the intestinal tissue perfusion (*T* = 0), 5 ROIs (Fig. 1) were marked and defined as follows: 2 on the lateral sides of the loop (well-perfused), 1 in the exact middle of the loop (not perfused), and 2 between the lateral and middle ROIs (partly perfused = watershed area). This method has been previously explained by our group [12]. Subsequently, a systemic lactate measurement was taken from the central ear vein. MB was injected after 10 min (*T* = 10) and bowel perfusion imaging in MB mode was performed for at least 60 s. Sixty minutes after ischemic loop creation, the camera system was switched to ICG mode, followed by ICG injection (*T* = 60) and fluorescence quantification analysis for 60 s. This procedure was directly followed by local capillary lactate sampling by puncturing the serosa at each of the 5 ROIs. The latter was done using a 23 Gauge needle and an EDGE lactate analyzer (ApexBio, Taipei, Taiwan, People’s Republic of China), which only requires a small drop of blood (3 μl) [12]. As lactate is a marker of ischemia [13], it was used as the gold standard to correlate the fluorescence signal. All animals were followed for a minimum of 120 min (*T* = 120). A schematic overview of the surgical procedure is displayed in Fig. 1.

### Fluorescence imaging system

The commercially available Quest Spectrum Fluorescence Camera (QUEST SPECTRUM®, Quest Medical Imaging, Middenmeer, The Netherlands) was used for NIRF imaging. The camera was fixed with a custom-made mechanical arm, which was connected to the surgical table to ensure a stable vision and distance to the ROI. The camera tip was fixed at 15 cm from the target organ in all operations. To avoid ambient light interference, all lights of the operating room were dimmed. As introduced, this camera system has bimodal properties and both channels were used in the current study; an 800 nm channel to capture ICG fluorescence (ICG mode) and a 700 nm channel for MB fluorescence imaging (MB mode). During the surgical procedure, four images were displayed on the screen (Supplementary S1). First, a standard color image of the surgical field was displayed alongside a grayscale NIRF image to aid surgical guidance. Moreover, two overlay modes were utilized, comprising a fluorescence intensity map and a NIRF image projected onto the colored image, all presented on the same screen.

### Statistical analysis and quantification

All NIRF images and videos were post-analyzed with Quest Spectrum software (ResearchTool v4.7, Quest Medical Imaging, Middenmeer, The Netherlands). As all ROIs were intraoperatively marked with a surgical marker, this was visible on all recordings and the exact same ROIs could be used during the analysis. A tracker synchronized the ROI with movement, and afterwards the software created a time-intensity curve of the measured intensity of the specific ROIs. The measured fluorescence intensity is displayed in arbitrary units (a.u.). Baseline subtraction was applied to all time-intensity curves. The ingress was the parameter used for the quantitative analysis of bowel perfusion assessment. The ingress quantifies the inflow in terms of increase in fluorescence intensity per second in the ROI (increase in a.u. per second: i/s). The ingress was calculated over a timeframe of 20 s after the end of the baseline.

Numerical variables were presented as medians with interquartile range (IQR). A Spearman’s rho was calculated to correlate local lactates with the fluorescence parameter. A *p* < 0.05 was considered significant. All statistical analyses were performed with the GraphPad Prism (GraphPad software for Apple, version 8.0.0, San Diego, California, United States).

## Results

A total of 4 pigs were included in this experiment (Table 1). Systemic lactate levels confirmed that there was no systemic ischemia during the experiment. No intraoperative dye-related complications occurred.

**Table 1:** Animal characteristics

	Pig 1	Pig 2	Pig 3	Pig 4
Weight (kg)	42.00	37.50	36.00	36.00
MB dose (mg/kg)	0.50	0.50	0.25	0.25
ICG dose (mg/kg)	0.20	0.20	0.20	0.20
Systemic lactate (mg/dL) *T10/T60*	19/23	15/23	18/21	12/14
Lactate ROI 1 *T10/T60*	21/19	15/8	19/17	12/12
Lactate ROI 2 *T10/T60*	28/57	40/41	16/16	59/53
Lactate ROI 3 *T10/T60*	102/73	36/68	50/84	43/69
Lactate ROI 4 *T10/T60*	34/91	42/46	15/8	43/51
Lactate ROI 5 *T10/T60*	24/22	17/26	15/17	12/26

*T* = time in minutes

### Bowel perfusion quantification

#### Time-intensity curves

**Fig. 2:**
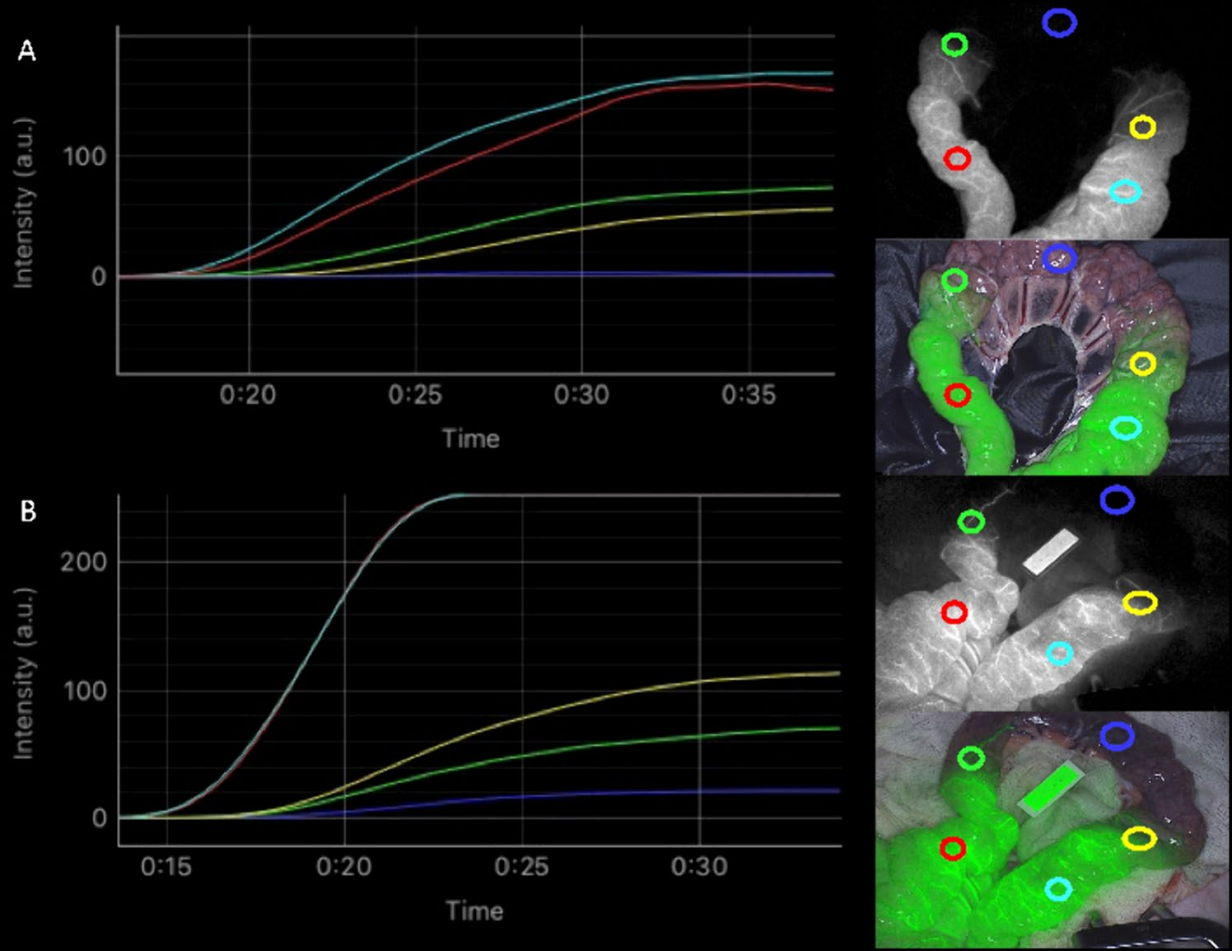
Time-intensity curve examples (Pig 3). ROI 1 = red (well-perfused), ROI2 = green (watershed), RO3 = blue (ischemic), ROI4 = yellow (watershed), ROI 5 = cyan (well-perfused), with corresponding NIRF images at 12 s. **A** MB mode (*T* = 10 min). **B** ICG mode (*T* = 60 min), red and cyanin line partly overlap (Color figure online)

In all included pigs, a clear macroscopic NIRF visualization of perfusion was achieved. For the well-perfused ROIs (1 and 5), the majority of curves displayed a steep ingress. In the time-intensity curves in the second ROI, marked as a watershed region, an inflow pattern comparable to ROI 4, also marked as a watershed region, was most often seen. For the ROIs with low perfusion (ROI 3), a clearly non-steep ingress and lower maximum fluorescence intensity is demonstrated compared to watershed and normal perfusion ROIs. An example of both MB and ICG time-intensity curves is displayed in Fig. 2.

#### Fluorescence quantification analysis: ingress correlation to lactate levels

**Fig. 3:**
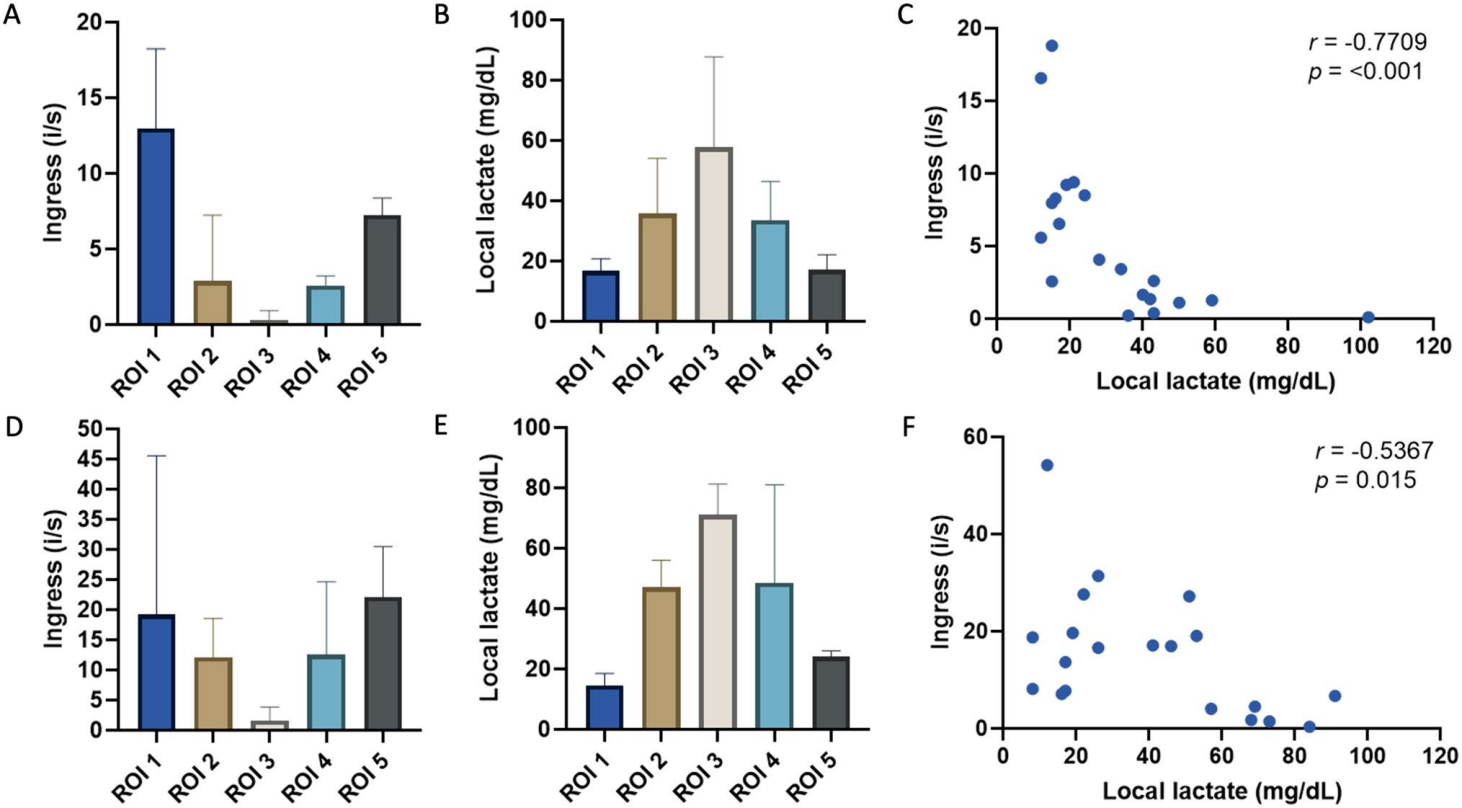
Results of bowel perfusion analysis. A-B-D-E values present medians, whiskers indicate the 75th percentile. **A** Ingress values of the 5 ROIs during MB administration (*T* = 10 min). **B** Local lactate levels of the 5 ROIs during MB administration (*T* = 10 min). **C** Scatterplot of ingress values and local lactate during MB administration showing a significant negative correlation (Spearman’s rho = − 0.7709, 95% CI − 0.9073 to − 0.4878; *p* =  < 0.001). **D** Ingress values for the 5 ROIs during ICG administration (*T* = 60 min). **E** Local lactate levels of the 5 ROIs during ICG administration (*T* = 60 min). **F** Scatterplot of ingress values and local lactate during ICG administration showing a significant negative correlation (Spearman’s rho = − 0.5367, 95% CI − 0.7965 to − 0.1096; *p* = 0.015). All detailed information is provided in Supplementary S2

First, all images of the ischemic loop during MB administration were analyzed (at *T* = 10 min). Ingress (i/s) values were calculated in all ROIs (Fig. 3A). ROIs 1 and 5 had a faster development of brightness as compared to ROIs 2, 3, and 4. It is also objectively proven with the fluorescence quantification analysis of the ingress in which ROI 3 had the lowest ingress. Compared to local lactate levels, the opposite patterns were seen; ROI 3 had lower levels of lactate compared to watershed and well-perfused areas (Fig. 3B). A Spearman’s correlation test showed a significant negative correlation between the ingress levels in the ischemic bowel loop and the corresponding local lactate levels (*r* = − 0.7709, 95% CI − 0.9073 to − 0.4878; *p* =  < 0.001) for MB fluorescence quantification analysis (Fig. 3C).

Secondly, the fluorescence quantification analysis of all images of the ischemic loop during ICG administration was performed (*T* = 60 min). All ROIs showed a similar development of increase in fluorescence intensity as during MB analysis (Fig. 3D). Compared to MB, absolute values were higher. Local lactate levels of ROI 3 also had lower levels compared to all other areas (Fig. 3E) and most of the values were higher than at *T* = 10 min. A second Spearman’s correlation test showed a significant negative correlation between ingress levels in the ischemic bowel loop and corresponding local lactate levels (*r* = − 0.5367, 95% CI − 0.7965 to − 0.1096; *p* = 0.015) for ICG fluorescence quantification analysis (Fig. 3F).

In one animal, NIRF imaging was performed of a non-ischemic bowel loop after injecting MB and ICG to compare absolute ingress values with the same method of administration, at the same timing. The ingress values were 7.02 i/s and 11.89 i/s for MB and ICG, respectively. No adverse reactions were observed in any of the animals after MB and ICG administration.

## Discussion

In this preclinical animal study, we have successfully performed a quantitative analysis of NIRF imaging for bowel perfusion using MB with a commercially available fluorescence imaging system. This analysis showed a significant negative correlation between local lactate levels (as a marker for ischemia) and MB ingress values. This correlation was stronger than the correlation for ICG quantification values, although the absolute ingress values of ICG were higher compared to MB. This camera system solved two significant drawbacks of MB as discussed in the previous literature [14]: (1) its absorption and emission in the vicinity of 700 nm, which is susceptible to increased background auto-fluorescence, and (2) the need for distinct equipment settings.

To imitate the clinical scenario involving bowel ischemia and/or inadequate anastomotic perfusion, we generated ischemic bowel loops in our experiment. As our goal was to validate fluorescence signals, we used lactate levels as an indicator of the perfusion state of the different ROIs [11, 13, 15]. It should be noted that some lactate levels decreased after one hour. A potential explanation could be linked to the existence of small overlapping vessels on the serosa, emanating from a neighboring intestinal segment with better perfusion. This setup might contribute to a slight reperfusion effect, a phenomenon recognized previously by Diana et al. [13]. For both MB and ICG imaging results, we found a significant negative Spearman’s correlation for the local lactate and corresponding ingress values in the same ROI. The results indicate that both dyes are suitable to assess bowel perfusion. Interestingly, the correlation of MB was stronger compared to the one of ICG (Spearman’s rho of − 7709 and − 0.5367, respectively). The latter may suggest that the use of MB for bowel perfusion assessment with this camera system may be more accurate. In contrast, we observed higher absolute ingress values for ICG imaging compared to MB. The finding that MB has a stronger correlation as opposed to ICG which shows higher absolute values may be attributed to the fact that ingress values were calculated at different time points (*T* = 10 min for MB and *T* = 60 min for ICG). To compare absolute ingress values, we performed NIRF imaging in one animal immediately after injecting MB and ICG into a non-ischemic bowel loop. The ingress values were 7.02 i/s for MB and 11.89 i/s for ICG, still indicating a slight difference. However, our research team considers the differences in the absolute values and correlation negligible as the real-time images obtained during surgery were very clear and informative for both dyes. We therefore do not state that one dye is better than the other, but we can conclude that MB may be as good as ICG for bowel perfusion assessment based on our quantitative analysis.

Adverse effects are important to consider when performing NIRF imaging with an optical dye. MB is a safe drug at a therapeutic dose below 2 mg/kg [9, 16], which is eight to four times higher than used in the current study (0.25 mg/kg and 0.5 mg/kg). There are some known adverse effects when administering doses above 2 mg/kg, such as hypertension, dyspnea, hemolysis, methemoglobinemia, nausea and vomiting, and pain in the chest [17], and it may precipitate serotonin toxicity if combined with other serotonergic drugs at doses above 5 mg/kg [18]. When levels are > 7 mg/kg, many of the adverse effects occur [16, 17, 19]. Refractory hypotension and skin discoloration are only known upon administration of 20 to 80 mg/kg [17], and anaphylactic reaction is extremely rare [9]. As the previous mentioned doses are much higher than needed for bowel perfusion assessment as demonstrated in this study, such adverse events are not expected for this indication. It is important to know that MB is contra-indicated together with serotonergic drugs, in glucose-6-phosphate dehydrogenase deficient patients, in patients with renal failure, and in pregnant women [9, 16]. Compared to ICG, MB has some more adverse reactions when administered in higher doses, but is currently completely safely used for visualization of thyroid and parathyroid glands, pancreatic neuroendocrine tumors, and breast cancer tumors and sentinel nodes within doses of < 2 mg/kg [9].

Although the use of ICG fluorescence is recommended in colorectal surgery to assess tissue perfusion [4], there is still no consensus on how to quantify fluorescence angiography. Previous studies were conducted to establish and gather validity evidence for a method of quantifying fluorescence angiography [20–24]. This revealed that bowel perfusion quantification is a feasible method to differentiate between different perfusion patterns, highlighting the possibility of using standardized imaging protocols [21]. According to a recent consensus paper on ICG fluorescence angiography, we concur with the authors’ standpoint that additional investigation into quantitatively evaluating fluorescence is imperative. This will help to reduce the subjective variability associated with perfusion assessment [21], and make it easier to compare study outcomes with different dyes, and will improve the validity and reproducibility of such data in daily practice.

The unique aspect of the present study is that, to our knowledge, no previous study has demonstrated the use of MB for bowel perfusion imaging in addition to the well-known and widely used ICG, within a single operative procedure and with a single commercially available NIRF imaging system. Based on the findings presented in this study and our previous investigations [8, 25], we can conclude that MB, when used in a dedicated imaging system, offers a range of simultaneous and multipurpose functionalities, all achieved solely through the administration of a single dose of MB. Based on several studies and recent consensus papers, the incorporation of ICG fluorescence for perfusion assessment during colorectal surgery has been shown to substantially decrease the risk of AL [2, 4, 14, 26]. It can even result in modifications to the resection line and/or adjustment of the anastomosis, and leads to shorter hospital stay and reduced overall morbidity [4]. ICG fluorescence highlights the added value of performing NIRF for bowel perfusion assessment. Considering that fluorescence imaging is not currently used in all medical facilities routinely, we anticipate that the findings from this study, along with our previous research, will encourage clinicians to explore the use of MB fluorescence. The advantage of using a single dye for multiple purposes makes it an appealing option for clinical practice.

## Limitations

There are some limitations in this animal study. The small sample size is a notable limitation. However, to ensure the feasibility of our hypothesis and adhere to the 3R principle (i.e., replace, reduce, refine) in animal research [27], the number of animals used in the study was considered adequate. Despite this small sample size, we believe that the correlations we present are sufficiently illustrative. Each pig underwent 5 measurements, resulting in a total of 20 measurements for each correlation analysis, which is sufficient for a Spearman correlation. Another limitation is that, although laparoscopic procedures are the norm for most elective abdominal clinical procedures, we used an open camera system in this study due to logistical constraints. Fortunately, there is a commercially available laparoscopic variant of the camera system used in our study. Additionally, while anastomotic perfusion is commonly required during colorectal resection and anastomosis creation, we used small bowel loops in our experiment. This decision was based on the challenges posed by the fixed and spiral orientation of a pig’s colon, making the small bowel a more suitable choice for illustrative purposes. It is essential to acknowledge that it differs from human colonic tissue, underscoring the need for human studies to provide crucial insights. Consequently, it is critical to interpret the current results cautiously, and human studies are necessary to assess the reproducibility of our findings in a clinical context.

## Conclusion

To conclude, we successfully performed a quantification analysis of a commercially available NIRF imaging system in this study. We demonstrated a significant negative correlation of ingress values of MB and ICG fluorescence quantification analysis with local lactate levels. This validates the potential to use MB for bowel perfusion assessment besides the well-known and widely used ICG. Further human studies are necessary to translate our findings to clinical applications.

### Supplementary Information

Below is the link to the electronic supplementary material.

Supplementary file1 (DOCX 1637 kb)
