# Supplementary material for: Quantitative analysis of intestinal perfusion with indocyanine green (ICG) and methylene blue (MB) using a single clinically approved fluorescence imaging system: a demonstration in a porcine model
Source: Surg Endosc. 2024 May 10;38(7):3556–63. doi: 10.1007/s00464-024-10864-1 (PMC11219451; doi:10.1007/s00464-024-10864-1)
Supplement: Supplementary file 1 — Supplementary file1 (DOCX 1637 kb) [file 464_2024_10864_MOESM1_ESM.docx]

**Supplementary - Quantitative analysis of intestinal perfusion with indocyanine green (ICG) and methylene blue (MB) using a single clinically approved fluorescence imaging system: a demonstration in a porcine model**

**S1.** Example of four displayed images/modes during surgery. Left upper display displays the standard color image of the surgical field; Right upper image displays a grayscale NIRF; left lower image displays overlay mode of fluorescence intensity map; right upper displays an overlay mode of the NIRF image projected onto the colored image.

**
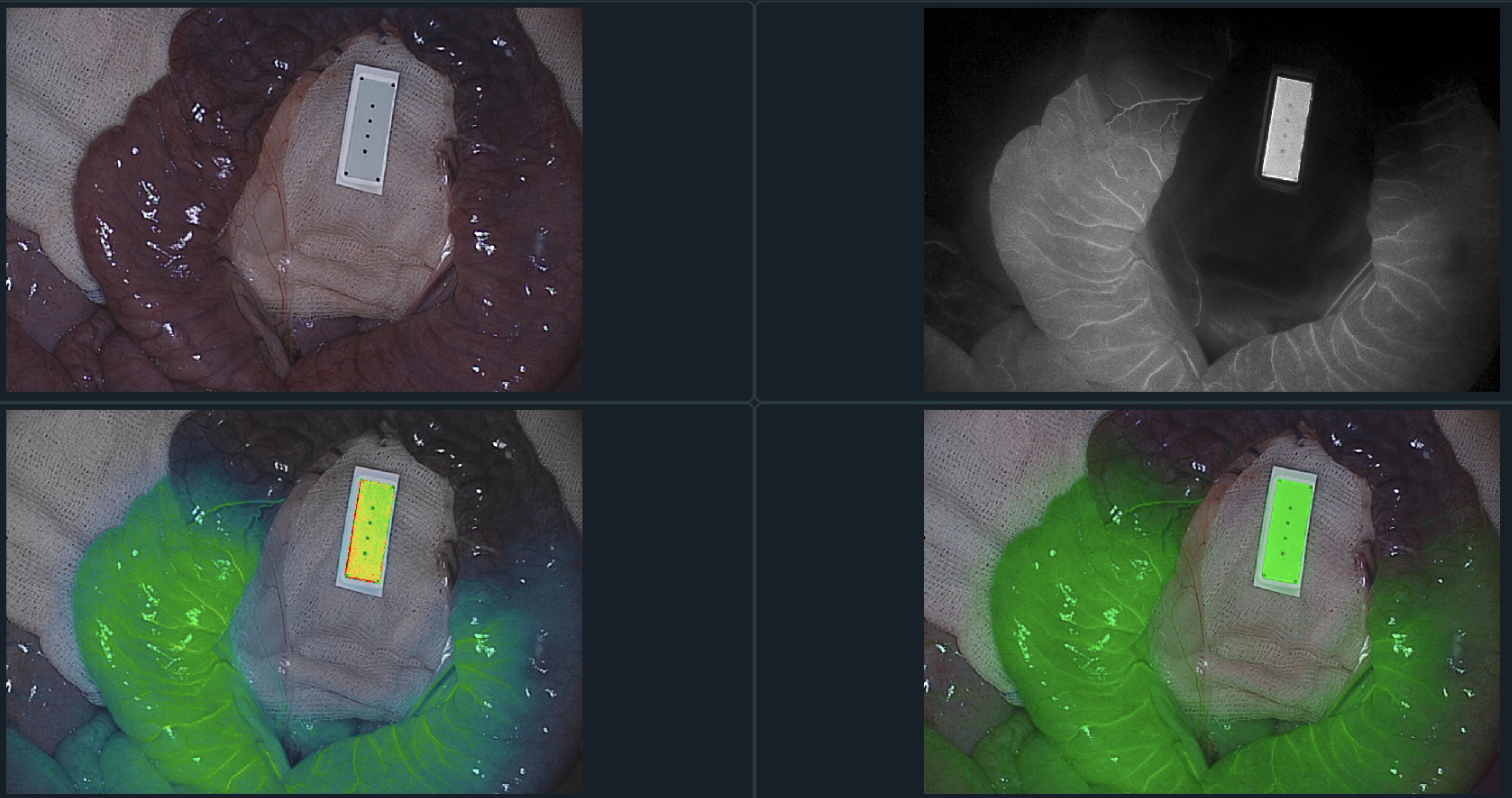
**

| **Region of interest** | **Lactate levels T10** | **Ingress values T10 (MB)** | **Lactate levels T60** | **Ingress values T60 (ICG)** |
| --- | --- | --- | --- | --- |
| ROI 1 | 21 | 9,41 | 19 | 19,66 |
| ROI 2 | 28 | 4,08 | 57 | 4,09 |
| ROI 3 | 102 | 0,1 | 73 | 1,47 |
| ROI 4 | 34 | 3,43 | 91 | 6,7 |
| ROI 5 | 24 | 8,51 | 22 | 27,62 |
| ROI 1 | 15 | 18,8 | 8 | 18,78 |
| ROI 2 | 40 | 1,66 | 41 | 17,1 |
| ROI 3 | 36 | 0,22 | 68 | 1,76 |
| ROI 4 | 42 | 1,35 | 46 | 16,97 |
| ROI 5 | 17 | 6,54 | 26 | 31,44 |
| ROI 1 | 19 | 9,22 | 17 | 13,7 |
| ROI 2 | 16 | 8,29 | 16 | 7,14 |
| ROI 3 | 50 | 1,11 | 84 | 0,36 |
| ROI 4 | 15 | 2,58 | 8 | 8,18 |
| ROI 5 | 15 | 7,97 | 17 | 7,76 |
| ROI 1 | 12 | 16,58 | 12 | 54,22 |
| ROI 2 | 59 | 1,27 | 53 | 19,07 |
| ROI 3 | 43 | 0,39 | 69 | 4,54 |
| ROI 4 | 43 | 2,61 | 51 | 27,2 |
| ROI 5 | 12 | 5,59 | 26 | 16,65 |

**S2.** Overview raw datapoints as presented in Figure 3 in the main paper.
